# How to Demonstrate Freedom from African Swine Fever in Wild Boar—Estonia as an Example

**DOI:** 10.3390/vaccines8020336

**Published:** 2020-06-25

**Authors:** Katja Schulz, Christoph Staubach, Sandra Blome, Imbi Nurmoja, Arvo Viltrop, Franz J. Conraths, Maarja Kristian, Carola Sauter-Louis

**Affiliations:** 1 Institute of Epidemiology, Friedrich-Loeffler-Institut, Federal Research Institute for Animal Health, Südufer 10, 17493 Greifswald, Insel Riems, Germany; Christoph.Staubach@fli.de (C.S.); Franz.Conraths@fli.de (F.J.C.); Carola.Sauter-Louis@fli.de (C.S.-L.); 2 Institute of Diagnostic Virology, Friedrich-Loeffler-Institut, Federal Research Institute for Animal Health, Südufer 10, 17493 Greifswald, Insel Riems, Germany; Sandra.Blome@fli.de; 3 Estonian Veterinary and Food Laboratory (VFL), Kreutzwaldi 30, 51006 Tartu, Estonia; Imbi.Nurmoja@vetlab.ee; 4 Estonian University of Life Science, Institute of Veterinary Medicine and Animal Sciences, Kreutzwaldi 62, 51014 Tartu, Estonia; Arvo.Viltrop@emu.ee; 5 Veterinary and Food Board, Väike-Paala 3, 11415 Tallinn, Estonia; maarja.kristian@vet.agri.ee

**Keywords:** African swine fever, disease freedom, wild boar, serology, surveillance

## Abstract

Estonia has been combatting African swine fever (ASF) for six years now. Since October 2017, the disease has only been detected in the wild boar population, but trade restrictions had to remain in place due to international regulations. Yet, the epidemiological course of the disease has changed within the last few years. The prevalence of ASF virus (ASFV)-positive wild boar decreased steadily towards 0%. In February 2019, the last ASFV-positive wild boar was detected. Since then, positive wild boar samples have exclusively been positive for ASFV-specific antibodies, suggesting the possible absence of circulating ASFV in the Estonian wild boar population. However, as the role of seropositive animals is controversially discussed and the presence of antibody-carriers is regarded as an indication of virus circulation at EU and OIE level, Estonia remains under trade restrictions. To make the disease status of a country reliable for trading partners and to facilitate the process of declaration of disease freedom, we suggest to monitor the prevalence of seropositive wild boar in absence of ASFV-positive animals. The possibility to include ASF in the list of diseases, for which an official pathway for recognition of disease status is defined by the OIE should be evaluated.

## 1. Introduction

African swine fever (ASF) emerged in Estonia in September 2014 for the first time. First cases were reported in wild boar and domestic pig farms were first affected by ASF outbreaks in 2015. The last ASF outbreaks in domestic pig holdings were notified in 2017 [1]. However, Estonia is still subject to trade restrictions and suffers economic losses as the country does not fulfil the criteria for disease freedom, although no ASFV-positive wild boar were detected since February 2019. Only the fact that there are still seropositive wild boar is taken as an indication for possible virus circulation in the population. With the exception of the island of Hiiumaa, where ASF has never been detected, the whole territory of Estonia has been designated as an infected zone and restrictions according to the Commission Implementing Decision of 9 October 2014 concerning animal health control measures relating to African swine fever in certain Member States (MS) and repealing Implementing Decision 2014/178/EU (2014/709/EU) apply (area with ASF occurrence in wild boar: Part II). Correspondingly, the dispatch of live pigs and pig by-products is restricted. By way of derogation, pigs can be dispatched to other areas and MS listed in part II or part III. However, the dispatch is accompanied by increased personal and financial resources (2014/709/EU Article 3). The exportation of live pigs in third countries is completely prohibited [2]. Also, the exportation of animal by-products is subject to strict regulations [2]. Within the EU, all listed regulations can be lifted as soon as a country shows the required evidence of disease freedom. However, the World Trade Organization (WTO) as well as third countries conduct their own risk assessments. Thus, third countries could impede the import of pigs and animal by-products based on their own risk assessment, even if the trade restrictions within the EU were lifted. A possibility to prevent arbitrary restrictions through third countries could be an official declaration by the OIE-Assembly. However, the procedure to gain official recognition of disease status by the OIE is currently reserved to seven defined diseases, which do not include ASF (https://www.oie.int/animal-health-in-the-world/official-disease-status/official-recognition-policy-and-procedures/, accessed 3 April 2020). 

Following the Commission Decision of 26 May 2003 approving an African swine fever diagnostic manual (2003/422/EC) and the current OIE Terrestrial Animal Health Code chapter 15.1.1, infection with ASF is present, when ASFV has been isolated from a suid sample or ASFV antigen identified in a sample or if ASFV-specific antibodies have been found in samples coming from domestic pigs or wild boar. The latter two definitions apply to samples, which originate from suids that showed clinical or pathological signs, were epidemiologically linked to ASF cases or were suspected to be associated with ASFV [2]. The current OIE definitions differ from the ones in the previous edition of the Terrestrial Animal Health Code, where the detection of ASFV-specific antibodies during the past 12 months was only considered as relevant in wild boar aged between 6 and 12 months [3]. 

Accordingly, a country can be considered free from ASF when no infection with ASF is observed and further criteria specified by the EU or the OIE are met. These include appropriate laboratory investigations, awareness programs, presence of competent veterinary authorities, sufficient surveillance activities, and the implementation of appropriate biosecurity measures [2]. No evidence has so far been found for the potential involvement of Ornithodoros ticks in the transmission and spread of ASF in Estonia [4]. Therefore, sufficient surveillance activities have to be shown for 12 months and the importation of pigs has to follow the regulations of the EU and the OIE before the country might be eligible to be declared free from ASF [2]. With regard to sufficient surveillance measures, the EU working document “Strategic approach to the management of African Swine Fever for the EU” (SANTE/7113/2015–Rev 11) recommends differentiating the surveillance activities between the aims of eradicating or controlling the disease. As Estonia aims to eliminate the disease, the focus is on both, enhanced passive surveillance, i.e., testing of all wild boar found dead for ASFV by PCR; and on active surveillance, i.e., testing all hunted wild boar for ASFV by PCR and for ASFV specific antibodies.

For areas or countries that are in the process of disease elimination, the current EU (2003/422/EC) and OIE recommendations prescribe the implementation of real-time PCR for virus detection and ELISAs for the detection of antibodies against ASFV as the standard procedure. As serological confirmation tests, IPT (indirect immunoperoxidase test), IBT (immune blotting test), and IFAT (indirect fluorescent antibody test) are recommended [5]. The respective standard operating procedures are provided by the European Union Reference Laboratory for ASF (https://asf-referencelab.info/asf/en/procedures-diagnosis/sops, accessed 14 May 2020) and their implementation in Estonia is described in detail elsewhere [6].

The case fatality ratio of ASF is known to be very high [7]. However, animals usually develop antibodies against ASFV within 7–10 days post infection, irrespective of the final disease outcome. They can be tested positive for ASFV and for antibodies simultaneously for at least 100 days after infection [8,9]. Samples from wild boar surviving this period are usually positive for ASFV-specific antibodies, but negative for ASFV and its genome [8,9,10]. Since a vaccine against ASF is not available [11,12], wild boar showing such test results more than 100 days post infection apparently survived the disease. 

It is not yet known for how long ASFV-specific antibodies persist and may be detected in wild boar; however, Penrith et al. [13] and Pujols Romeu et al. [14] found them several years after infection. Penrith et al. [13] also found ASFV-specific maternal antibodies in piglets younger than six months of age. Although these findings refer to domestic pigs, one can assume that the situation in wild boar is similar. While diaplacental virus transmission to the fetus can occur after infection with Classical Swine Fever (CSF) virus [15,16], for ASF, scientific evidence for intrauterine infection is scarce and inconclusive [8,17,18,19,20,21].

The role of surviving animals in the spread or in the maintenance of ASF is still controversially discussed. In a recent study, it was hypothesized that survivors with virus persisting in lymphatic tissues could start shedding virus when stressed or immunocompromised [22]. However, there is no scientific evidence that survivors really serve as carriers and could thus pose a risk for maintaining the disease in the population [9,10,23,24].

Recent analyses of Estonian wild boar surveillance data showed a clear decline of wild boar samples that were ASFV-positive by PCR. Even in areas where the first cases were not detected before 2016, the prevalence of PCR-positive wild boar started to decrease by the end of 2017. At the same time, an increase of seropositive, but PCR-negative test results was observed. Furthermore, in areas, where the epidemic had started in 2014 and 2015 (Southern and Northeastern part of the country), the initially increasing seroprevalence already started to decrease in July 2018 [10]. Since March 2019, all positive ASF samples have been exclusively serologically positive, i.e., showing ASFV-specific antibodies, although all surveillance activities in Estonia are still performed in accordance with the regulations for an infected country and intensive surveillance is implemented. 

In a previous study, data for the period December 2014–July 2018 was analyzed to investigate the course of ASF in the East and the West of Estonia [10]. Based on this first investigation, the presented study broadens the scope by including recent surveillance data and assesses the situation in the whole of Estonia. Based on our findings, we propose adapting the current EU and OIE regulations, taking the latest scientific results into account [9,10,23,24,25]. Due to the presumably long half-life of ASFV-specific antibodies [13,14], seropositive wild boar may be present for several years without constituting a risk of disease transmission. In contrast to CSF, which could be controlled by oral vaccination in wild boar [26], it is of utmost importance to explore alternatives to re-gain ASF-free status without vaccination. To protect countries like Estonia from individual trade restrictions through third countries, we propose not only defining the current EU and OIE requirements more precisely, but to add ASF to the list of diseases, for which official recognition of disease status can be granted by the OIE. These changes may support elimination efforts in other countries, where the epidemic of ASF seems to run a similar course [25] and could acknowledge the global importance, ASF has gained in recent years. 

## 2. Materials and Methods

### 2.1. Data

Wild boar surveillance data from the whole of Estonia that covered a period from January 2015 until February 2020 were used (62 study months, 6 study years). The data were entered by the Estonian veterinary authorities into the CSF/ASF wild boar surveillance database of the European Union (https://surv-wildboar.eu), from which they were obtained with the approval of the Estonian authorities for the analyses described here. The structure of the data set, the sampling process and the laboratory investigations are described in detail elsewhere [6]. Samples originated either from active (hunted wild boar) or passive surveillance (wild boar that were found dead, shot due to sickness, or killed in a road traffic accident). Each data record contained information about the virological (PCR) and the serological (ELISA and for confirmation IPT) test result. Blood samples were taken from hunted wild boar that were tested for ASFV genome and for ASFV-specific antibodies. From animals found dead, tissue or bone marrow samples were collected and investigated for ASFV genome [6]. All samples were processed following the Diagnostic Manual valid in the EU (2003/422/EC).

All data records lacking a conclusive virological test result (i.e., neither positive nor negative) (*n* = 135) or information on the origin of sample (active or passive surveillance) (*n* = 2) were excluded. Estonia is divided into 15 counties, which represented the basis of our analyses. Due to an administrative reform in 2017, all data records from 2015–2017 were assigned to the new administrative counties.

Population data from the Estonian Environment Agency (Nature department) were used to investigate the trend of the wild boar population density in Estonia in the context of ASF. The analysis continued those performed by Schulz et al. [10] by adding data from the hunting seasons of 2018/19 and 2019/20 to those from the hunting seasons 2012/13–2017/18. Data was provided on county level and the estimated number of wild boar per km^2^ was calculated for the whole of Estonia. The detailed description of the data has previously been provided [6]. 

### 2.2. Prevalence Analyses

Prevalence estimates were calculated for PCR-positive wild boar (irrespectively of their serological test result), for wild boar that were PCR and serologically positive and for wild boar with a PCR-negative, but a positive serological test result, in the same way as described by Schulz et al. [10] and Oļševskis et al. [25]. The prevalence estimates were calculated for the whole of Estonia, for each age class (younger than one year, between one and two years, and older than two years) and for each of the 62 study months. Prevalences were estimated using the software package R (http://www.r-project) [27] and 95% confidence intervals were computed according to Clopper and Pearson [28]. 

### 2.3. Modeling Temporal Effect

Model analysis was applied to data from wild boar samples that had yielded a conclusive serological test result (negative or positive) and were PCR-negative. The temporal course of the raw seroprevalence estimates was calculated for each study month using a Bayesian space–time model and BayesX 2.0.1 (http://www.uni-goettingen.de/de/bayesx/550513.html). A detailed description of the model and the variables is provided elsewhere [10,29]. In brief, age was defined as a fixed independent variable. In contrast to the prevalence estimations, age was categorized into two classes (animals younger than two years and animals older than two year) for the model analysis. The seroprevalence was defined as a dependent variable and time, space, and season were included as random factors. From the original data set, all data records with a positive PCR result were removed (*n* = 2936). Also, samples from passive surveillance (*n* = 672), samples without information for age (*n* = 547) and samples lacking a conclusive serological test result (*n* = 82) were excluded for the model analysis. All figures were generated using the software package R (http://www.r-project) [27].

### 2.4. Population Density

The number of wild boar per km^2^ in all 15 counties of Estonia were summarized and analyzed for the whole country. Differences between the population densities in the hunting seasons were analyzed as described previously [10]. A *p*-value of <0.05 was considered as statistically significant.

## 3. Results

### 3.1. Data

After excluding all data records with invalid information, 46,093 data records were available. In the 62 study months, the number of samples originating from hunted wild boar was clearly higher than the number of those coming from passive surveillance. The fluctuations in the sample size followed a clear seasonal pattern with the highest number of samples taken during winter. This applies to samples from both active and passive surveillance, likewise (Figure S1 Supplemental Material). The largest numbers of samples (14,947 samples from active surveillance and 978 samples from passive surveillance) were obtained in 2016. In 2018 and 2019, the numbers of samples were clearly lower in both categories. However, in 2020, where information was so far only available for the first two months, already 1346 samples from active surveillance were investigated and 10 samples originated from passive surveillance (Table 1). 

### 3.2. Prevalence Analyses

**Figure 1:**
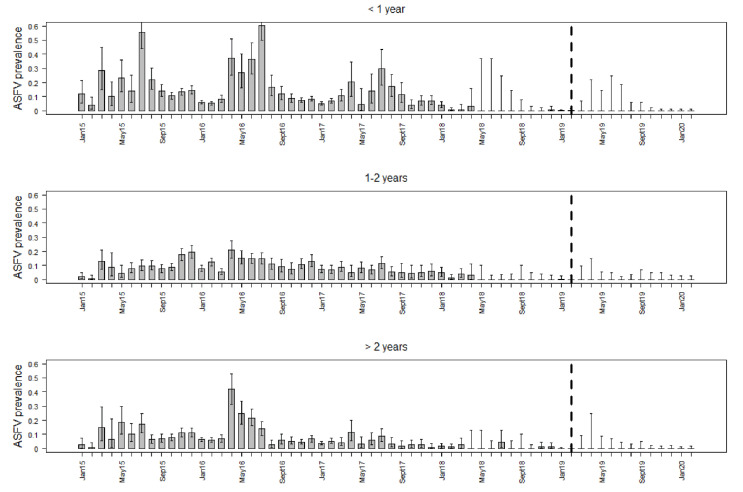
Prevalence estimates for ASFV PCR-positive wild boar, irrespective of their serological status for each study month and three different age classes. The whiskers indicate 95% confidence intervals. The broken vertical line highlights February 2019, when ASFV-positive wild boar were last detected by PCR.

Of all three prevalence groups (PCR-positive, only seropositive or positive for ASFV and ASFV-specific antibodies), PCR-positive wild boar showed the highest prevalence estimates, whereby the prevalence estimates resulted in the highest values in animals younger than one year (Table 2). The ASFV prevalence estimates in wild boar younger than one year were higher in spring and summer (Figure 1). The PCR-prevalence decreased over time and reached 0%, where it remained since March 2019 in all three age classes (Figure 1).

**Figure 2:**
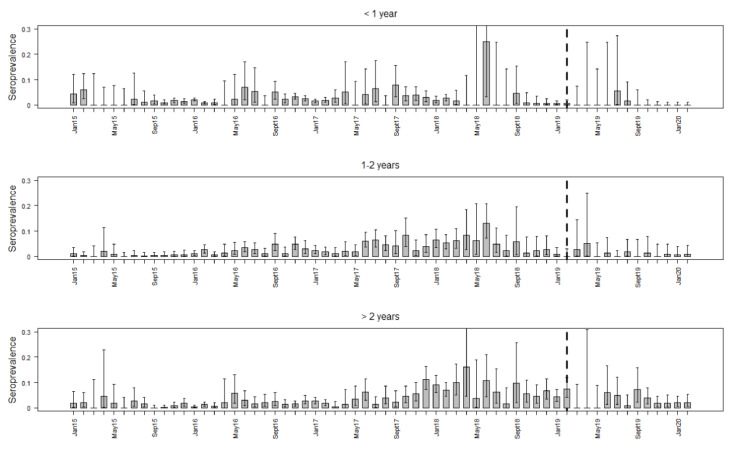
Prevalence estimates for seropositive wild boar that were ASFV PCR-negative for each study month and three different age classes. The whiskers indicate 95% confidence intervals. The broken vertical line highlights February 2019, when ASFV-positive wild boar were last detected by PCR.

The seroprevalence was clearly lower in all three age classes than the ASFV prevalence (Table 2). Especially in animals older than two years, an increase in the seroprevalence over time was observed. During the last six months of the study period, the seroprevalence decreased in all three age classes, but particularly in wild boar younger than one year. In this age class, the last two seropositive wild boar were detected in July and August 2019 (Figure 2).

**Figure 3:**
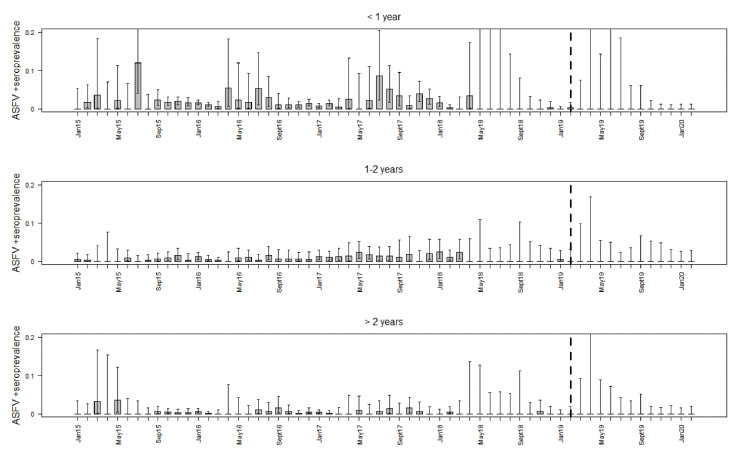
Prevalence estimates for ASFV PCR-positive and seropositive wild boar test results for each study month and three age classes. The whiskers indicate 95% confidence intervals. The broken vertical line highlights February 2019, the last month, in which ASFV-positive wild boar were detected.

The prevalence of wild boar that tested positive for both, ASFV-specific antibodies and ASFV by PCR were even lower than the seroprevalence estimates (Table 2). The lowest values were observed in wild boar older than two years. From May 2018 onwards, the prevalence estimates were extremely low in all three age classes, with a 0% prevalence in the last 12 (younger than one year), the last 13 (between 1 and 2 years), or the last 15 months (older than 2 years) of the study period, respectively (Figure 3). 

### 3.3. Modeling Temporal Effect

**Figure 4:**
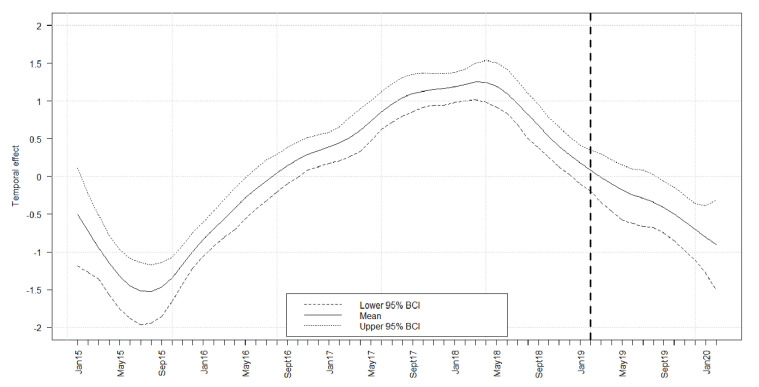
Median temporal effect on the logit prevalence for all samples that tested exclusively serologically positive, 95% Bayesian credible intervals (BCI) are indicated. The broken vertical line highlights February 2019, when the last ASFV-positive wild boar were detected by PCR.

For the model analysis of the temporal effect on the prevalence, 41,856 data records were used. Adjusted for seasonal effects (Figure S2 Supplemental Material), the temporal course of seroprevalence estimates was investigated. Until April 2018, an increase in the temporal trend of the logit prevalence of seropositive wild boar was observed. Subsequently, the logit prevalence decreased steadily until the end of the study period (Figure 4). 

### 3.4. Population Density

The wild boar population in Estonia was stable from 2012/13 until 2015/16 with no significant difference between the single hunting seasons. However, the population density dropped statistically significantly (*p*-value = 0.015) from hunting season 2015/16 to hunting season 2016/17. Also from 2016/17 to 2017/18, the population density decreased, but the difference was not statistically significant (Figure S3 Supplemental Material). From hunting season 2016/17 onwards, the reported number of wild boar and the calculated number of wild boar per km^2^ was highest on the island of Hiiumaa, where no ASF cases have occurred so far (Table S1 Supplemental Material). 

## 4. Discussion

In Estonia, the last ASFV PCR-positive wild boar was detected in February 2019. In December 2018 and January 2019, only nine ASFV-positive wild boar were detected, from which eight were found close to the eastern border of the country. Most likely, these cases were epidemiologically linked to the ASF situation in the western part of the Russian Federation, where ASFV circulation was reported between September and November 2018 (http://www.fsvps.ru/fsvps/asf, accessed 7 March 2019).

Since then, all cases reported as ASF-positive were ASFV-negative by PCR and only seropositive. According to EU regulation 2003/422/EC and the current Terrestrial Animal Health Code of the OIE [2], it makes no difference for the status of the country, if animals are ASFV-positive, i.e., virus positive; or seropositive (if an epidemiological link to ASFV cases exists), i.e., the country remained under trade restrictions [2]. With regard to the seropositive test results, the judgement concerning the presence of an epidemiological link to ASFV-positive cases is left to the individual countries, but it will be difficult for a country like Estonia to argue that the seropositive cases found there are not epidemiologically linked to previous ASFV infections in wild boar. Yet, the regulation leaves already some room for interpretation, while it was stricter in the former OIE Code text, where seropositive test results were only considered as relevant, if they originated from wild boar aged between six and 12 months [3]. 

In the current ASF epidemic, which started in Georgia in 2007 [30], the Czech Republic provided the documents to the OIE in support of a self-declaration of re-gaining an ASF-free status. This declaration must not be confused with the official recognition of disease status by the OIE (https://www.oie.int/animal-health-in-the-world/official-disease-status/official-recognition-policy-and-procedures/, accessed 3 April 2020). 

In accordance with the requirements of the OIE [2], the Czech Republic declared the freedom from ASF in April 2019, after one year of the last confirmed ASFV-positive cases in wild boar (https://www.oie.int/fileadmin/Home/eng/Animal_Health_in_the_World/docs/pdf/Self-declarations/2019_05_CzechRep_ASF_ANG.pdf, accessed 3 April 2020). In this self-declaration of the Czech Republic, data from the time of the epidemic (26 June 2017 till 28 February 2019) has been presented (https://www.oie.int/fileadmin/Home/eng/Animal_Health_in_the_World/docs/pdf/Self-declarations/2019_05_CzechRep_ASF_ANG.pdf, accessed 3 April 2020). No information is provided about the kind of investigations performed (virologically or serologically) in this document. In an official presentation, more detailed data have been shown, indicating that serologically positive hunted wild boar were sampled in July and October 2018 (http://web.oie.int/RREurope/eng/Regprog/docs/docs/SGE%20ASF12/BTSF/02_Sampling_and_laboratory_testing_Pavel_Bartak.pdf, accessed 22 April 2020). This indicates the presence of seropositive wild boar only few months before the country submitted the self-declaration of freedom from ASF to the OIE. However, the seropositive animals may not have originated from wild boar younger than one year and thus, the Czech Republic may not have considered them as directly linked to a suspected or confirmed case of ASF. Therefore, these findings were not regarded as contradicting the requirements of the OIE for a self-declaration of freedom from ASF [2]. Moreover, no new cases of ASF were so far detected in the Czech Republic, suggesting the absence of ASFV circulation in the country, despite the potential presence of seropositive wild boar.

In the majority of countries that are affected by the current ASF epidemic, infected wild boar populations play a major role. This experience contrasts with previous African swine fever epidemics in Europe, which mainly involved domestic pigs and were often under control after a few outbreaks had occurred [31]. After the successful implementation of control measures, the disease status was resolved. On the Iberian Peninsula, however, the situation was more complicated and the disease became endemic for decades. In 1995, when Spain was officially declared as free from ASF in domestic pigs, wild boar and their ASF status were not included in the statement [31,32]. Perez et al. [33], who detected a seroprevalence of almost 10% in wild boar in some areas of Spain, recommended repeating serological investigations after the declaration of freedom, but wild boar were not considered as a relevant reservoir for ASFV at that time and trade restrictions regarding Spain were ultimately lifted [34]. Although Mur et al. [31] found many years later no ASFV or ASFV-specific antibodies, the seroprevalence in Spanish wild boar was unknown in 1995.

This example from the past, the ambiguity of the self-declaration of disease freedom, the temporal trend of ASF test results in Estonia, the continuous huge surveillance effort in countries like Estonia and the lack of scientific evidence for the existence of carrier animals and their obvious negligible epidemiological role, if they existed [9,23], were the motivation to initiate the present study. The aim was to analyze ASF surveillance data with regard to the temporal trend of the ASFV prevalence and seroprevalence and to evaluate surveillance activities. The results lead us to propose a revision of the current EU and OIE definitions regarding the freedom from ASF status or even adding ASF to the diseases that can gain an official recognition of disease status by the OIE.

The decrease in the sample size over time correlates most probably with the decreasing population density. Particularly regarding the reduced numbers of samples originating from passive surveillance, decreased chances to detect a dead wild boar can be assumed due to the lower population density and the reduced circulation of ASF. Following the working document of the EU “Strategic approach to the management of African Swine Fever for the EU” (SANTE/7113/2015–Rev 11), the number of wild boar carcasses detectable declines in the phase of a decreasing epidemic. This supports the hypothesis that the ASF epidemic in Estonia is subsiding. However, the effort to find dead wild boar and to sample every detected wild boar, thus performing surveillance according to the EU (SANTE/7113/2015–Rev 11) and to the Article 15.1.30 to 15.1.32 of the OIE Terrestrial Code [2] is of course still high and must remain so for the foreseeable future to detect any re-incursion or re-emergence as soon as possible. 

The study results also show a clear seasonality in the number of ASF samples. In the months of hunting season (October–February), the numbers of samples are higher, particularly the ones taken from hunted wild boar. Also the number of samples originating from passive surveillance are higher in the winter months, which may be due to the less dense vegetation and the increased presence of hunters in the forests. However, to increase the certainty in the assessment of freedom from ASFV, more efforts should be made to increase the sample size also in times of usually low hunting activities. This is particularly important as the results of this study suggest a high ASFV prevalence in the summer months. Although high ASFV prevalence estimates were mainly found in wild boar younger than one year, which is likely to be due to the generally high case fatality ratio in young animals, these results are in accord with observations from other affected countries [24], even further emphasizing the need to raise sampling efforts in the summer months (risk-based sampling). 

The seroprevalence, which increased until May 2018 in Estonia [10], has since decreased steadily, whereas the ASFV prevalence as measured by PCR has remained at a point estimate of 0% for more than 12 months. In Latvia, a similar course of disease can currently be observed [25]. In Sardinia, where the disease has been endemic since 1978, a recent survey yielded a very high mean seroprevalence above 50% and a low mean ASFV prevalence (2.6%) in free-ranging pigs [35]. Between 2015 and 2018, intensive control measures were applied in Sardinia, including the elimination of free ranging pigs, which are known to play an important role in the spread of ASF on the island [36]. Thus, this positive course might be the beginning of disease elimination. However, a continued endemic situation of the disease in Sardinia cannot be excluded at this stage. In Estonia, however, only seropositive wild boar have been found since more than 12 months, which may suggest that ASFV is no longer circulating and the epidemic might fade out, if there is no new introduction or re-emerging infection from an yet undetected focus. The latter seems unlikely in view of the intensity and spatial representativeness of the surveillance activities in Estonia. 

The last seropositive wild boar younger than one year was detected in August 2019. Unfortunately, age is only classified as younger than one year, between one and two years and as older than two years in the records of the surveillance data. It is therefore not possible to tell if the seropositive wild boar that was hunted in August 2019 was between one and six months of age or between 6 and 12 months [37,38]. This increases the uncertainty regarding the determination of the time interval, when the last ASFV infections occurred in Estonia.

According to the definitions of the previous OIE Code, Estonia does therefore not fulfil the requirements for a self-declaration of freedom from ASF before September 2020. The slight changes in the previous and the current definitions probably resulted from the debate regarding the possible existence of carrier animals and their potential role in ASFV spread [23]. In addition, the differentiation between these two age classifications in the previous OIE Code was probably due to the assumption that maternal antibodies may be present in wild boar younger than six months [13,19,37,39]. As long as ASFV is not detected in such animals, indicating that no new infection has occurred, it can be assumed that seropositive young animals do not pose a risk for the spread the disease. By contrast, seropositive wild boar between 6 and 12 months could have been infected several months prior to sampling, suggesting ASFV circulation within the previous months. However, EU regulations and the current edition of the OIE Code do not include the above-mentioned age distinction anymore. It rather considers seropositive wild boar of all ages as relevant as long as they show clinical or pathological signs of an ASFV infection or are epidemiologically linked to ASF cases [2]. However, due to the lack of the differentiation between animals younger or older than six months, we propose to re-evaluate this definition. If no ASFV-positive wild boar have been detected for at least one year, seropositive animals between one and two years might still suggest circulating ASFV, so that a disease-free status is not warranted. By contrast, isolated seropositive animals younger than one year may be present due to maternal antibodies. These animals would not increase the risk of disease spread. At the same time, wild boar older than two years are old enough to have become infected at the time, when ASFV was still circulating. They have obviously cleared the infection as they are PCR-negative for ASFV and do therefore not pose a risk of spreading the disease. 

Previously, the evolution of attenuated ASFV strains that could complicate detection and control has been suggested [8,40,41]. Indeed, a virus strain with reduced virulence was detected in Estonia. However, this could only be demonstrated regionally and over a short period of time [8]. Despite intensive searching for the attenuated virus with a tailored PCR, it could not be found in any of the samples originating from the affected region in the following years. However, even the potential circulation of such a strain would not change the role of seropositive animals, i.e., animals, which survived infection. Experimental data do not suggest a higher risk for carriers or chronically infected animals [40]. To include the risk of the potential circulation of attenuated virus strains in surveillance activities, the course of the seroprevalence has to be evaluated carefully. A sudden increase of seropositive animals without concurrent increase of ASFV-positive animals may indicate the presence of an attenuated ASFV strain and thus require more intensive testing. So far, this has not been the case. Although an accumulation of seropositive animals was observed over time, there was no significant peak after May 2018.

The potentially long half-life time of ASFV-specific antibodies [13,14] and the resulting accumulation of seropositive wild boar support the hypothesis that the ASFV circulation has come to an end in Estonia. The presumed accumulation of seropositive animals has probably led to the presented study results, which show a higher seroprevalence in older animals, particularly at the end of the study period. This has also been observed in Latvia [25]. When this knowledge about the potential long half-life and accumulation of seropositive wild boar is taken into account in the evaluation of surveillance data, a long time period can be expected, during which seropositive wild boar might be detected [37]. Therefore, a more precise definition regarding the role of seropositive, but ASFV-negative wild boar during the late phase of an ASF epidemic is needed, as the current regulations may impede the self-declaration of freedom from ASF status in a country in a way that is not warranted by scientific evidence. One way to define the disease status of a country more clearly might be to attribute wild boar only to two age categories, namely animals younger than two years and those older than two years. The vague transition period between young and sub-adult wild boar [38,42,43] could thus be avoided and more accurate epidemiological conclusions could be drawn based on the laboratory test results and the age of the animals. Finally, the possibility to provide a self-declaration of disease freedom, including the inconclusive definition of the existence of an epidemiological link, does not support a clear and reliable statement about the disease status of a country. The consideration of a more precise definition could clearly help to increase the certainty for potential trading partners. 

## 5. Conclusions

In Estonia, no ASFV has been detected for more than one year now. At the same time, strong biosecurity measures, surveillance activities, and ongoing awareness campaigns are in place. The wild boar population density decreased significantly, probably due to a combination of ASFV circulation and intensified hunting. This low population density supports the control of ASF and should therefore be maintained. In addition, surveillance activities have to be retained, possibly focusing on risk-based sampling. The last few ASFV-positive cases emphasize in particular the increased need to sample in risk areas close to the external borders of Estonia. Moreover, sampling should be increased during summer months and younger wild boar should be investigated for ASFV and ASFV-specific antibodies, while the testing of older animals for ASFV-specific antibodies should be paramount. If Estonia upholds its strong efforts to detect any new introduction as early as possible and to monitor the age and the current course of seropositive wild boar continuously, the country should be able to submit a self-declaration regarding the recovery of freedom from ASF in due course. In the case of attained disease freedom, surveillance activities must be maintained so that early detection of any re-introduction or re-emergence is warranted. Freedom from disease must be repeatedly demonstrated. To circumvent the uncertainties regarding the status of freedom from ASF status, we propose to add ASF to the list of diseases for which an official procedure for recognition of disease status by the OIE already exists. This may decrease potential risks in the international trade and help to prevent trade restrictions that are not based on scientific evidence. 

## Figures and Tables

**Table 1:** Numbers of investigated wild boar samples for each year of the study period from active (i.e., from hunted animals) and from passive surveillance (i.e., from animals found dead, shot due to sickness or killed in road traffic accidents)

	Year	2015	2016	2017	2018	2019	2020 *
Number of Samples							
**Active surveillance**		8580	14,947	9150	4884	4715	1346
**Passive surveillance**		941	978	401	79	62	10
**Total**		9521	15,925	9551	4963	4777	1356

* For 2020, only data from the first two months were included.

**Table 2:** Median, minimum, and maximum prevalence estimates for wild boar of three different age classes for the period January 2015–February 2020. The animals had tested positive by PCR, were seropositive or positive for ASFV, and had ASFV-specific antibodies.

Age Classes	Prevalence ASFV (PCR-Positive)			Prevalence Seropositive			Prevalence ASFV- (PCR-) and Seropositive		
	**Median**	**Min**	**Max**	**Median**	**Min**	**Max**	**Median**	**Min**	**Max**
**<1 year**	0.057	0.000	0.602	0.011	0.000	0.250	0.007	0.000	0.119
**1–2 years**	0.051	0.000	0.209	0.019	0.000	0.130	0.003	0.000	0.025
**>2 years**	0.027	0.000	0.419	0.022	0.000	0.160	0.000	0.000	0.035

## Supplementary Materials

The following are available online at https://www.mdpi.com/2076-393X/8/2/336/s1, Figure S1: Number of all investigated wild boar samples for each study month starting in January 2015 and ending in February 2020. The numbers refer to samples originating from active surveillance (i.e., from hunted animals; white bars) and from passive surveillance (i.e., from animals found dead, shot due to sickness or killed in road traffic accidents; black bars); Figure S2: Median seasonal effect of all samples that tested exclusively serologically positive on the logit prevalence. 95% Bayesian credible intervals (BCI) are indicated. The broken vertical line highlights February 2019, the last month, in which ASFV-positive wild boar were detected; Figure S3: Wild boar population density (number of wild boar/km^2^) in Estonia during the study period (hunting seasons 2012/2013; 12/13, to 2019/2020; 19/20) The horizontal line that forms the top of the box marks the 75th percentile, the line that forms the bottom the 25th percentile. The horizontal line that intersects the box indicates the median number of wild boar per square kilometer. Whiskers represent the maximum and minimum values that were within the 1.5 times span of the interquartile range. Open circles represent outliers, i.e., single values greater or smaller than the extremes indicated by the whiskers, Table S1: Number of wild boar per km^2^ in the counties of Estonia in the hunting seasons 2012/2013–2019/2020.
