# Supplementary material for: How to Demonstrate Freedom from African Swine Fever in Wild Boar—Estonia as an Example
Source: Vaccines (Basel). 2020 Jun 25;8(2):336. doi: 10.3390/vaccines8020336 (PMC7350251; doi:10.3390/vaccines8020336)
Supplement: Supplementary file 1 [file vaccines-08-00336-s001.pdf]

vaccines

Supplementary File

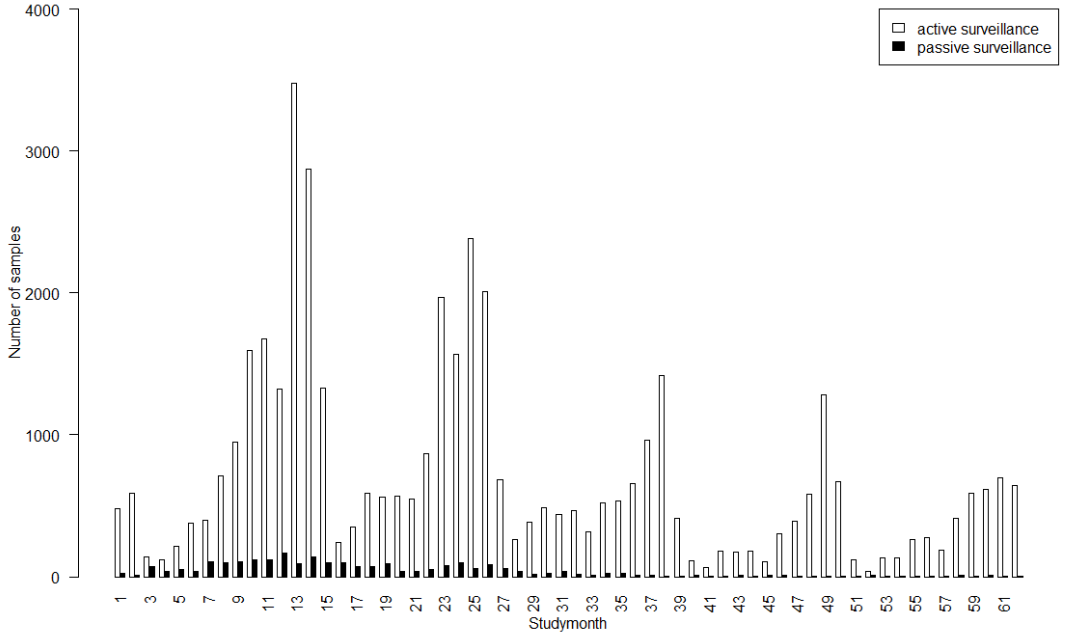

Figure S1. Number of all investigated wild boar samples for each study month starting in January 2015 and ending in February 2020. The numbers refer to samples originating from active surveillance (i.e. from hunted animals; white bars) and from passive surveillance (i.e. from animals found dead, shot due to sickness or killed in road traffic accidents; black bars).

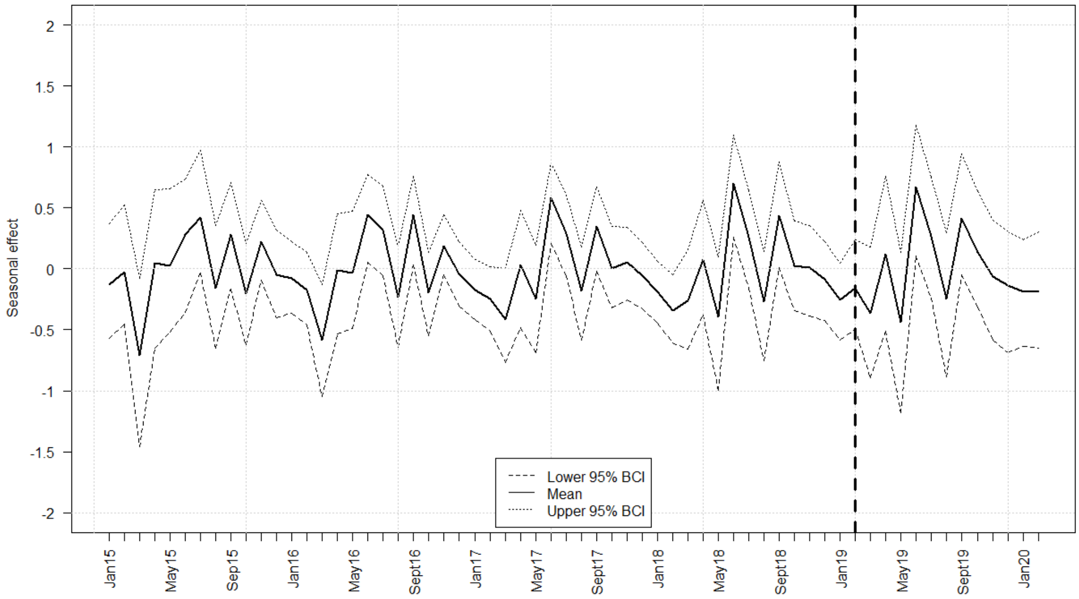

Figure S2. Median seasonal effect of all samples that tested exclusively serologically positive on the logit prevalence. 95% Bayesian credible intervals (BCI) are indicated. The broken vertical line highlights February 2019, the last month, in which ASFV-positive wild boar were detected.

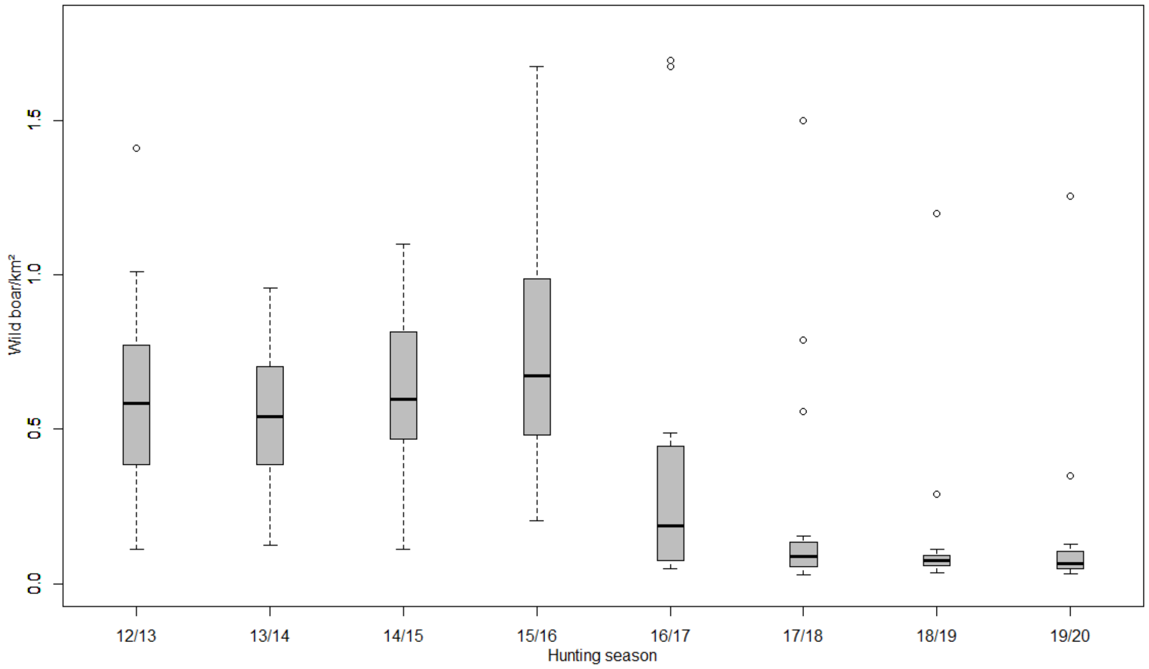

Figure S3. Wild boar population density (number of wild boar/km²) in Estonia during the study period (hunting seasons 2012/2013; 12/13, to 2019/2020; 19/20) The horizontal line that forms the top of the box marks the 75th percentile, the line that forms the bottom the 25th percentile. The horizontal line that intersects the box indicates the median number of wild boar per square kilometer. Whiskers represent the maximum and minimum values that were within the 1.5 times span of the interquartile range. Open circles represent outliers, i.e. single values greater or smaller than the extremes indicated by the whiskers.

Table S1. Number of wild boar per km^2 in the counties of Estonia in the hunting seasons 2012/2013 - 2019/2020.

Hunting season / County	2012/13	2013/14	2014/15	2015/16	2016/17	2017/18	2018/19	2019/20
**Harjumaa**	0.347	0.315	0.416	0.631	0.380	0.155	0.073	0.053
**Hiiumaa**	0.992	0.831	0.888	1.475	1.979	1.500	1.199	1.256
**Ida-Virumaa**	0.111	0.125	0.111	0.205	0.188	0.085	0.087	0.064
**Jõgevamaa**	0.346	0.381	0.479	0.672	0.117	0.028	0.036	0.033
**Järvamaa**	0.751	0.674	0.798	0.894	0.111	0.053	0.069	0.074
**Läänemaa**	1.009	0.735	0.986	1.674	1.695	0.557	0.088	0.046
**Lääne-Virumaa**	0.385	0.417	0.601	0.855	0.275	0.048	0.038	0.050
**Põlvamaa**	0.795	0.758	0.831	0.805	0.050	0.057	0.055	0.093
**Pärnumaa**	0.477	0.390	0.469	0.619	0.401	0.089	0.059	0.040
**Raplamaa**	0.616	0.557	0.756	1.080	0.488	0.096	0.059	0.050
**Saaremaa**	1.409	0.958	1.100	1.965	1.673	0.790	0.290	0.351
**Tartumaa**	0.390	0.373	0.542	0.541	0.075	0.093	0.094	0.066
**Valgamaa**	0.594	0.549	0.391	0.270	0.064	0.066	0.075	0.100
**Viljandimaa**	0.537	0.449	0.471	0.364	0.054	0.056	0.091	0.112
**Võrumaa**	0.584	0.540	0.597	0.420	0.073	0.113	0.113	0.127
